# Average volume-assured pressure support for patients with obstructive sleep apnea with failed CPAP titration

**DOI:** 10.5935/1984-0063.20210015

**Published:** 2022

**Authors:** Naomitsu Watanabe, John M Levri, Victor T Peng, Steven M Scharf, Montserrat Diaz-Abad

**Affiliations:** 1 University of Maryland School of Medicine, Medicine - Baltimore - Maryland - United States.; 2 University of Maryland Medical Center Midtown Campus, Medicine - Baltimore - Maryland - United States.

**Keywords:** Obstructive Sleep Apnea, AVAPS, Therapy, Refractory, CPAP, Noninvasive Ventilation

## Abstract

**Objectives:**

Obstructive sleep apnea (OSA) is a common disease, often treated using continuous positive airway pressure (CPAP) therapy. In many cases, patients fail a CPAP titration study due to inadequate control of the apnea-hypopnea index (AHI, events/hour) or due to treatment-emergent central sleep apnea (TE-CSA). We report our experience using a mode of non-invasive ventilation for alternative treatment of these patients.

**Material and Methods:**

We reviewed records of adults who had OSA with AHI≥15 diagnosed on polysomnography (PSG) with failed CPAP titration and in whom titrations with average volume-assured pressure support (AVAPS) with auto-titrating expiratory positive airway pressure were performed.

**Results:**

Forty-five patients, age 57.9±13.1 y, 26 males, body mass index (BMI) 40.2±8.7kg/m^2^. Reasons for CPAP titration failure included: TE-CSA (25, 55.6%) and inadequate control of AHI at maximum CPAP of 20cm H2O (20, 44.4%). Changes noted from baseline PSG to AVAPS titration: AHI: 65.3±29.3 decreased to 22.3±16.1 (p<0.001). Median time SpO2 ≤88%: 63.7 to 6.9min (p<0.001). In 16 patients the AHI was reduced to <15 and in 16 additional patients the AHI was reduced to <30. Improvement in AHI was not related to gender, age, or opioid use, but was correlated with BMI: ∆AHI=12.2 - (1.4 * BMI); p=0.05. AVAPS resulted in improved sleep architecture: median N3 sleep increased: 1.4% to 19.6% total sleep time (TST) (p<0.001), and median R sleep increased: 6.4% to 13.6% TST (p<0.01).

**Discussion:**

For patients with OSA for whom CPAP titration failed, titration with AVAPS may be an effective treatment.

## INTRODUCTION

Obstructive sleep apnea (OSA) is a common disease, with estimated prevalence of 3% and 10% in women and men, respectively, between ages 30 to 49 years, and 9% and 17% in women and men, respectively, between ages 50 to 70 years^1^. Continuous positive airway pressure (CPAP) is the most common standard treatment option. The CPAP pressure level is usually determined with an in-laboratory titration study or with at-home treatment using auto-titrating CPAP. An optimal manual CPAP titration is defined as that leading to an apnea-hypopnea index (AHI, events/hour) being ≤5 for 15 minutes^2-5^. However, some patients undergoing CPAP titration “fail” either due to the development of treatment-emergent central sleep apnea (TE-CSA) during titration or to inadequate control of the AHI over the range of CPAP pressures to the maximum used^6-9^. Reasons for failure include increased nasal resistance^10^, obesity, and daytime hypoxia/hypercapnia^11^. While the prevalence of such CPAP titration failure is unknown, there are few evidence-based guidelines on how to manage CPAP treatment failure. There are other positive airway pressure (PAP) modalities that have shown some effectiveness in CPAP titration failures. These include: bilevel positive airway pressure in spontaneous mode (bilevel PAP) for pressure intolerance; adaptive servo-ventilation (ASV); and bilevel positive airway pressure in spontaneous-timed mode (bilevel PAP ST) for TE-CSA^12-25^. Currently, with increased mortality reported in a large randomized controlled trial in patients with heart failure with reduced ejection fraction, ASV is generally not considered one of the more common treatment options^20-26^.

Average volume-assured pressure support (AVAPS) is a recently developed advanced bilevel PAP ST non-invasive ventilation (NIV) mode^27^. This mode adjusts inspiratory positive airway pressure (IPAP) to achieve a target tidal volume. AVAPS can be applied either with a constant level of expiratory positive airway pressure (EPAP), or with auto-titrating EPAP (AVAPS-AE). Thus, the ventilatory assist mode adjusts pressures to achieve a target tidal volume and will adjust the pressure regimen depending on the patient’s ventilatory pattern, and also can adjust the EPAP level to treat OSA. There is at least one case report showing that AVAPS could in fact function as a rescue modality in severe OSA that failed CPAP therapy^28^. Another study showed iVAPS (intelligent volume-assured pressure support - essentially equivalent to AVAPS) with auto-titrating EPAP was equivalent for control of disordered breathing events compared to iVAPS using a fixed EPAP level pre-determined from an in-laboratory titration, in patients with hypoventilation and concomitant OSA^29^.

In this study, we examined the efficacy of using AVAPS in patients with documented OSA in whom in-laboratory CPAP titration studies were not successful in bringing about satisfactory control of the disease. We hypothesized that in patients with OSA in whom in-laboratory CPAP titration studies were unsuccessful there would be noted improvement during an AVAPS titration study.

## MATERIAL AND METHODS

### Patient selection

The University of Maryland Institutional Review Board approved this retrospective medical records review. Adults (age ≥18 years) who had an AVAPS-AE titration study performed from January 2014 to October 2019 for failure of CPAP to treat OSA during an in-laboratory titration study were included. All patients had standard in-laboratory full night polysomnography (PSG) documenting the presence of OSA based on standard diagnostic criteria from the International Classification of Sleep Disorders - third edition^30^. All patients had an unsuccessful prior attempt at CPAP titration on a second in-laboratory PSG.

Medical records were reviewed to gather information regarding demographics, including age, gender, body mass index (BMI), comorbidities (including hypertension, chronic heart failure, chronic lung disease, among others), the indications for AVAPS titration based on the preceding CPAP titration study, and Epworth Sleepiness Scale.

### In-laboratory PSG

All in-laboratory sleep studies were attended by registered licensed registered polysomnographic technologists (RPSGT) and were performed in an academic American Academy of Sleep Medicine (AASM) - accredited sleep laboratory. Studies included: full-night diagnostic PSG, CPAP titration, and AVAPS-AE titration. All studies were scored by a RPSGT and interpreted by board-certified sleep medicine specialists based on standard criteria^31^.

Apneas were defined as a reduction in the peak signal excursion by ≥90% of the pre-event baseline lasting for at least 10 seconds. Obstructive apneas were defined as apneas with continued or increased respiratory effort, whereas central apneas were defined as absence of respiratory effort during absent airflow. A mixed apnea was scored if it was associated with absent inspiratory effort in the initial portion of the event, followed by resumption of inspiratory effort in the latter portion of the event. The unit of measurement was the AHI, defined as the sum of apneas plus hypopneas per hour of sleep. Hypopneas were scored per the two accepted definitions. Both of these define hypopneas as meeting all of the following: (a) the peak flow signal excursion drops by ≥30% of pre-event baseline using nasal air pressure (diagnostic study) or PAP device flow (titration study), and (b) the duration of the ≥30% drop in signal excursion is ≥10 seconds. For AHI4, at least a 4% reduction in oxygen saturation is required in the hypopnea. For AHI3, a 3% reduction in oxygen saturation or a terminal arousal is required in the hypopnea^31,32^.

### Baseline PSG

Data from the initial diagnostic PSG were gathered including AHI3 and AHI4, oxygenation variables (minimum oxygen saturation, time oxygen saturation ≤88%), and variables concerning sleep architecture including time in sleep stages N1, N2, N3, and R, total sleep time, sleep latency, and R latency.

### CPAP titration PSG

CPAP titration was performed using the AASM guidelines on manual titration of CPAP^5^. Patients in whom CPAP titration was considered a “failure” were those in whom the AHI did not fall below 15 at the highest level of CPAP used in the laboratory (20cm H_2_O), or those who developed TE-CSA that constituted >50% of the final AHI during the CPAP titration.

### AVAPS titration PSG

An in-laboratory AVAPS study was performed within 4 weeks of the CPAP titration attempt, using the OmniLab Advanced +, System One device (Philips Respironics, Murrysville, PA, U.S.), with the following default settings: minimal EPAP: 4cm H_2_O, maximal EPAP: 14cm H_2_O, minimal pressure support (PS): 4cm H_2_O, maximal PS: 21cm H_2_O, maximal pressure: 25cm H_2_O, AVAPS rate: 2, inspiratory time: 1.5 seconds, tidal volume: 8ml/kg ideal body weight, and breath rate: 12/minute. All AVAPS studies were performed with the auto-titrating EPAP (AE) function.

### Outcome measures

We compared the various outcomes between initial diagnostic PSG and AVAPS titration study. AVAPS’ effectiveness in reducing AHI, improving oxygenation, and improving measures of sleep architecture were target end points. Effective treatment was defined as achieving an AHI <15 in the AVAPS titration study.

### Statistical analysis

Data were collected and collated. Normality was determined using the Shapiro-Wilk test. Normally distributed data were expressed as mean ± standard deviation, while non-normally distributed data were expressed as median (interquartile distance). Statistical significance between means was determined using t-test for paired or unpaired variates as appropriate (normal distribution). For non-normally distributed data, differences between medians were tested using a Sign-Rank test or Sign-rank Sum test as appropriate. To compare differences between baseline PSG, CPAP results, and AVAPS, we used repeated measures analysis of variance for normally distributed data and repeated measures analysis of ranks for non-normally distributed data. Differences between proportions were investigated using chi-square testing. Association between variables was determined using linear regression. Statistical significance was determined at the 5% level. SigmaPlot version 14 (Systat software, 2017, San Jose, CA, U.S.) was used to perform statistical analysis.

## RESULTS

During the period studied, 2,550 CPAP titration studies were performed. A total of forty-five patients who met the inclusion criteria above were included: mean age was 57.9±13.1 years, with 26 (57.8%) males. Table 1 shows the demographic data as well as comorbid conditions. The mean BMI was elevated, and the most prevalent (>30%) comorbidities included hypertension, congestive heart failure, coronary artery disease, chronic lung disease, diabetes, and hyperlipidemia. The reasons for CPAP titration failure and indications for an AVAPS titration study included: TE-CSA (n=25, 55.6%), and failure of maximal tolerated CPAP pressure (up to 20cm H_2_O) to effectively treat OSA (n=20, 44.4%).

**Table 1:** Baseline characteristics and comorbidities.

Variable	Values (n=45)
Age (y)	57.9±13.1
Gender	26M, 19F
Body mass index (kg/m^2^)	40.2±8.7
Epworth Sleepiness Scale	10.7±7.9
Hypertension	40 (88.9)
Congestive heart failure	17 (37.8)
Coronary artery disease	14 (31.1)
Atrial flutter/fibrillation	8 (17.8)
Pulmonary hypertension	6 (13.3)
Chronic lung disease	17 (37.8)
Cerebrovascular disease	1 (2.2)
Chronic kidney disease	5 (11.1)
Diabetes mellitus	22 (48.9)
Opioid use	9 (20)
Hyperlipidemia	22 (48.9)
Restless legs syndrome	2 (4.4)
Depression disorder	8 (17.8)
Gastro-esophageal reflux disease	10 (22.2)
Bipolar disorder	1 (2.2)
Anxiety disorder	1 (2.2)
Insomnia disorder	1 (2.2)
Post-traumatic stress disorder	1 (2.2)
Narcolepsy	1 (2.2)

**Notes:** Data shown as mean ± standard deviation or n (% of total).

Table 2 shows the results of baseline PSG and the effects of AVAPS on indices of OSA severity. AVAPS titration was associated with significant improvement in AHI3 and AHI4, indices of oxygenation, as well as time in R and N3 sleep. The AHI4 was reduced from 54.3±23.2 to 19.1±6.1, *p*<0.001, and the AHI3 was reduced from 65.3±29.3 to 22.3±16.1, *p*<0.001.

**Table 2:** Comparison between diagnostic PSG, CPAP, and AVAPS.

Measurements	PSG	CPAP	AVAPS	p*	p**
AHI4 (events/h)	54.3±23.2	42.5±25.9	19.1±6.1	<0.001	0.006
AHI3 (events/h)	65.3±29.3	43.3±24.4	22.3±16.1	<0.001	<0.001
Time oxygen saturation ≤88% (min)	63.7 (3.6, 139.2)	66.6 (0.5, 140.8)	6.9 (1.3, 63.6)	<0.001	NS
Total sleep time (min)	299 (221.0, 362)	295.6 (247.6, 350.8)	309.5 (280.8, 347.8)	NS	NS
Sleep efficiency (%)	72.9 (56.7, 82.4)	70.3 (59.4, 86.3)	77.3 (66.0, 85.6)	NS	NS
N3 (%)	1.4 (0.0, 15.4)	10.8 (0.3, 18.4)	19.6 (4.5, 44.0)	<0.001	NS
R (%)	6.4 (4.1, 14.4)	10.7 (5.5,14.4)	13.6 (7.1, 17.5)	0.008	NS
Minimum oxygen saturation (%)	74 (62, 85)	73.8 (71, 87)	81 (70, 86)	0.015	NS
Sleep latency (min)	20.1 (8.5, 52.5)	28.7 (7.3, 44.0)	14 (6.5, 31.0)	NS	NS
R latency (min)	159.5±108.9	129.4+74.7	129.8±98.3	NS	NS

* Comparison of PSG with AVAPS;

** Comparison of PSG with CPAP; Data shown as mean ± standard deviation for normally distributed data, or median (interquartile distance) for non-normally distributed data. **Abbreviations:** PSG = Polysomnography; CPAP = Continuous positive airway pressure; AVAPS = Average volume-assured pressure support; AHI4 = Apnea-hypopnea index including hypopneas with at least 4% reduction in oxygen saturation; AHI3 = Apnea-hypopnea index including hypopneas with a 3% reduction in oxygen saturation or a terminal arousal.

Sixteen (35.6%) patients achieved an AHI<15 on AVAPS and 16 additional patients (35.6%) had an AHI <30. Thus, the number of patients with AHI <30 was 7 (16%) on baseline PSG, while on AVAPS, this number increased to 32 (71.1%). Improvement in AHI was not related to gender, age, or opioid use, but was correlated with BMI. For AHI4, the regression equation was ∆AHI4=24.2 - (1.6*BMI), adjusted R^2^=b0.12, *p*=0.012. For AHI3, the regression equation was ∆AHI3=12.2 - (1.38*BMI); adjusted R^2^=0.121; *p*=0.011.

## DISCUSSION

In this study we demonstrate that AVAPS led to improvement in the severity of OSA in many patients for whom CPAP titration did not successfully treat OSA, either due to failure to treat disordered breathing events during CPAP titration, or due to the development of TE-CSA. In the ensuing discussion, we consider these findings in light of the currently available literature.

For patients with OSA, CPAP is generally regarded as the most effective therapy for reduction of AHI, although it is thought that adherence could affect the magnitude of therapeutic effect. CPAP therapy has a significant beneficial effect in patients with OSA, with a recent meta-analysis suggesting a cardiovascular mortality benefit^13^. An attended in-laboratory titration is considered the gold standard for initiation of CPAP therapy; however, a sizable minority of these titration studies end up being suboptimal, per established criteria^5^. While some studies indirectly reported the incidence of CPAP titration failure as 28-50%^33-34^, there are few studies that comprehensively reported on the incidence of each cause of CPAP failure. TE-CSA has been most extensively studied, which is estimated to occur in 1-15% of patients with OSA^35-41^.

Additionally, there are few guidelines on management of CPAP titration failure, although commonly used modalities include bilevel PAP for pressure intolerance, ASV and bilevel PAP ST for TE-CSA. Bilevel PAP has been advocated as a rescue therapy by several groups in the setting of poor CPAP adherence or CPAP intolerance^20^. One recent study^20^, which used auto-bilevel PAP reported a substantial improvement of AHI for both CPAP-intolerant and TE-CSA groups. In another study, bilevel PAP ST improved the AHI in patients with CPAP failure^23^. However, in this study, minimal oxygen saturation at baseline of the patient population was considerably higher at 92.9±1.8% than our study population, which had a minimum oxygen saturation of 74% (62, 85), suggesting that our patients had more severe underlying cardiopulmonary disease.

A recent large randomized controlled study showed that ASV was more effective in reducing AHI and improving oxygenation than CPAP^25^. In this study, sleep architecture variables were not provided. Additionally, a recent study in heart failure patients demonstrated increased mortality with ASV^26^. As 37.8% of our patients had a known comorbidity of congestive heart failure, it is likely that ASV may not be recommended for use in many patients as rescue treatment.

Our study is one of the first to evaluate the effectiveness of AVAPS treatment in OSA with CPAP treatment failure during all-night in-laboratory titration. Given that CPAP treatment failure can lead to many patients with OSA in whom treatment cannot be offered, the results suggest that AVAPS may be an effective alternative. Consistent with our results, a case report describing very severe OSA in a pediatric patient with CPAP treatment failure showed that AVAPS successfully treated the condition, avoiding tracheostomy^28^.

In our study, we demonstrated that BMI was the only factor correlated with the magnitude of the improvement in AHI (∆AHI); the greater the BMI, the less the effect of AVAPS relative to baseline PSG. These results agree with previous studies showing that obesity is associated with greater CPAP titration failure rate^11^. The reasons for this remain unclear. However, the critical closing pressure (passive), which represents the tendency for the upper airway to collapse, is known to be correlated with BMI^43-45^, and is thought to mediate this correlation. The larger the BMI, the the greater the tendency was for the upper airway to collapse, and the smaller the expected benefit of AVAPS. Unfortunately, we did not have data on daytime PaO_2_ or PaCO_2_ in most of our patients. It has been reported that patients with lower PaO_2_ and higher PaCO_2_ are more likely to develop TE-CSA^11^.

This study suggests the efficacy of AVAPS in patients meeting our inclusion criteria. Larger studies should be carried out to determine specific criteria for using AVAPS in patients with CPAP titration failure. A further limitation of this study is that this was a retrospective review of a single center’s experience with a small number of patients. Larger multicenter trials could allow a larger number of patients using a greater range of settings. A strength of this study is that the included cases consisted of a varied range of demographics and comorbidities that would also be seen in the general population.

## CONCLUSION

For patients with OSA for whom CPAP titration failed, titration with AVAPS was an effective rescue treatment option for many patients. More studies are needed to determine the role of this NIV mode in patients with OSA who fail a traditional CPAP titration.

## References

[1] Peppard PE, Young T, Barnet JH, Palta M, Hagen EW, Hla KM (2013). Increased prevalence of sleep-disordered breathing in adults. Am J Epidemiol.

[2] Veasey SC, Rosen IM (2019). Obstructive sleep apnea in adults. N Engl J Med.

[3] Patel SR (2019). Obstructive sleep apnea. Ann Intern Med.

[4] Gottlieb DJ, Punjabi NM (2020). Diagnosis and management of obstructive sleep apnea: a review. JAMA.

[5] Kushida CA, Chediak A, Berry RB, Brown LK, Gozal D, Iber C (2008). Clinical guidelines for the manual titration of positive airway pressure in patients with obstructive sleep apnea. J Clin Sleep Med.

[6] Morgenthaler TI, Kagramanov V, Hanak V, Decker PA (2006). Complex sleep apnea syndrome. Is it a unique clinical syndrome. Sleep.

[7] Hoffman M, Schulman DA (2012). The appearance of central sleep apnea after treatment of obstructive sleep apnea. Chest.

[8] Kuziniar TJ, Morgenthaler TI (2012). Treatment of complex sleep apnea syndrome. Chest.

[9] Javaheri S, Smith J, Chung E (2009). The prevalence and natural history of complex sleep apnea. J Clin Sleep Med.

[10] Nakata S, Noda A, Yagi H, Yanagi E, Mimura T, Okada T (2005). Nasal resistance for determinant factor of nasal surgery in CPAP failure patients with obstructive sleep apnea syndrome. Rhinology.

[11] Kuklisova Z, Tkacova R, Joppa P, Wouters E, Sastry M (2017). Severity of nocturnal hypoxia and daytime hypercapnia predicts CPAP failure in patients with COPD and obstructive sleep apnea overlap syndrome. Sleep Med.

[12] Kushida CA, Littner MR, Hirshkowitz M, Morgenthaler TI, Alessi CA, Bailey D (2006). Practice parameters for the use of continuous and bilevel positive airway pressure devices to treat adult patients with sleep-related breathing disorders. Sleep.

[13] Patil SP, Ayappa IA, Caples SM, Kimoff RJ, Patel SR, Harrod CG (2019). Treatment of adult obstructive sleep apnea with positive airway pressure: an American Academy of Sleep Medicine systematic review, meta-analysis, and GRADE assessment. J Clin Sleep Med.

[14] Kuźniar TJ, Morgenthaler TI (2012). Treatment of complex sleep apnea syndrome. Chest.

[15] Sanders MH, Kern N (1990). Obstructive sleep apnea treated by independently adjusted inspiratory and expiratory positive airway pressures via nasal mask. Physiologic clinical implications. Chest.

[16] Gentina T, Fortin F, Douay B, Dernis JM, Herengt F, Bout JC (2011). Auto bi-level with pressure relief during exhalation as a rescue therapy for optimally treated obstructive sleep apnoea patients with poor compliance to continuous positive airways pressure therapy - a pilot study. Sleep Breath.

[17] Blau A, Minx M, Peter JG, Glos M, Penzel T, Baumann G (2012). Auto bi-level pressure relief-PAP is as effective as CPAP in OSA patients - a pilot study. Sleep Breath.

[18] Gay PC, Herold DL, Olson EJ (2003). A randomized, double-blind clinical trial comparing continuous positive airway pressure with a novel bilevel pressure system for treatment of obstructive sleep apnea syndrome. Sleep.

[19] Ballard RD, Gay PC, Strollo PJ (2007). Interventions to improve compliance in sleep apnea patients previously non-compliant with continuous positive airway pressure. J Clin Sleep Med.

[20] Carlucci A, Ceriana P, Mancini M, Cirio S, Pierucci P, Lupo ND (2015). Efficacy of bilevel auto-treatment in patients with obstructive sleep apnea not responsive to or intolerant of continuous positive airway pressure ventilation. J Clin Sleep Med.

[21] Allam JS, Olson EJ, Gay PC, Morgenthaler TI (2007). Efficacy of adaptive servoventilation in treatment of complex and central sleep apnea syndromes. Chest.

[22] Brown SE, Mosko SS, Davis JA, Pierce RA, Godfrey-Pixton TV (2011). A retrospective case series of adaptive servoventilation for complex sleep apnea. J Clin Sleep Med.

[23] Morgenthaler TI, Gay PC, Gordon N, Brown LK (2007). Adaptive servoventilation versus noninvasive positive pressure ventilation for central, mixed, and complex sleep apnea syndromes. Sleep.

[24] Westhoff M, Arzt M, Litterst P (2012). Prevalence and treatment of central sleep apnoea emerging after initiation of continuous positive airway pressure in patients with obstructive sleep apnoea without evidence of heart failure. Sleep Breath.

[25] Morgenthaler TI, Kuzniar TJ, Wolfe LF, Willes L, McLain WC, Goldberg R (2014). The complex sleep apnea resolution study: a prospective randomized controlled trial of continuous positive airway pressure versus adaptive servoventilation therapy. Sleep.

[26] Cowie MR, Woehrle H, Wegscheider K, Angermann C, D’Ortho MP, Erdmann E (2015). Adaptive servoventilation for central sleep apnea in systolic heart failure. N Engl J Med.

[27] Piper AJ (2020). Advances in non-invasive positive airway pressure technology. Respirology.

[28] Diaz-Abad M, Isaiah A, Rogers VE, Pereira KD, Lasso-Pirot A (2018). Use of noninvasive ventilation with volume-assured pressure support to avoid tracheostomy in severe obstructive sleep apnea. Case Rep Pediatr.

[29] McArdle N, Rea C, King S, Maddison K, Ramanan D, Ketheeswaran S (2017). Treating chronic hypoventilation with automatic adjustable versus fixed EPAP intelligent volume-assured positive airway pressure support (iVAPS): a randomized controlled trial. Sleep.

[30] American Academy of Sleep Medicine (AASM) (2014). International classification of sleep disorders.

[31] Berry RB, Gamaldo CE, Harding SM, Brooks R, Lloyd RM, Vaughn BV (2015). The AASM manual for the scoring of sleep and associated events.

[32] Wahba N, Sayeeduddin S, Diaz-Abad M, Scharf SM (2019). The utility of current criteria for split-night polysomnography for predicting CPAP eligibility. Sleep Breath.

[33] Schiza SE, Bouloukaki I, Mermigkis C, Panagou P, Tzanakis N, Moniaki V (2011). Utility of formulas predicting the optimal nasal continuous positive airway pressure in a Greek population. Sleep Breath.

[34] Gupta A, Shukla G (2018). Polysomnographic determinants of requirement for advanced positive pressure therapeutic options for obstructive sleep apnea. Sleep Breath.

[35] Javaheri S, Smith J, Chung E (2009). The prevalence and natural history of complex sleep apnea. J Clin Sleep Med.

[36] Morgenthaler TI, Kagramanov V, Hanak V, Decker PA (2006). Complex sleep apnea syndrome: is it a unique clinical syndrome?. Sleep.

[37] Kuźniar TJ, Kasibowska-Kuźniar K, Ray DW, Freedom T (2013). Clinical heterogeneity of patients with complex sleep apnea syndrome. Sleep Breath Schlaf Atm.

[38] Cassel W, Canisius S, Becker HF, Leistner S, Ploch T, Jerrentrup A (2011). A prospective polysomnographic study on the evolution of complex sleep apnoea. Eur Respir J.

[39] Khan MT, Franco RA (2014). Complex sleep apnea syndrome. Sleep Disord.

[40] Lehman S, Antic NA, Thompson C, Catcheside PG, Mercer J, McEvoy RD (2007). Central sleep apnea on commencement of continuous positive airway pressure in patients with a primary diagnosis of obstructive sleep apnea-hypopnea. J Clin Sleep Med.

[41] Liu D, Armitstead J, Benjafield A, Shao S, Malhotra A, Cistulli PA (2017). Pepin JL. Trajectories of emergent central sleep apnea during CPAP therapy. Chest.

[42] Rowley JA, Tarbichi AG, Badr MS (2005). The use of a predicted CPAP equation improves CPAP titration success. Sleep Breath.

[43] Younes M (2019). Pathogenesis of obstructive sleep apnea. Clin Chest Med.

[44] Pham LV, Schwartz AR (2015). The pathogenesis of obstructive sleep apnea. J Thorac Dis.

[45] Genta PR, Schorr F, Eckert DJ, Gebrim E, Kayamori F, Moriya HT (2014). Upper airway collapsibility is associated with obesity and hyoid position. Sleep.

